# Straightforward Patterning of Functional Polymers by Sequential Nanosecond Pulsed Laser Irradiation

**DOI:** 10.3390/nano11051123

**Published:** 2021-04-27

**Authors:** Edgar Gutiérrez-Fernández, Tiberio A. Ezquerra, Aurora Nogales, Esther Rebollar

**Affiliations:** 1 Instituto de Estructura de la Materia, IEM-CSIC, Serrano 121, 28006 Madrid, Spain; edgar.gutierrez@ehu.eus (E.G.-F.); t.ezquerra@csic.es (T.A.E.); aurora.nogales@csic.es (A.N.); 2 Instituto de Química Física Rocasolano, IQFR-CSIC, Serrano 119, 28006 Madrid, Spain

**Keywords:** laser-induced periodic surface structures, polymer nanostructures, ordered nanostructures, linear gratings, nanodots arrays, semiconductor polymer, ferroelectric polymer

## Abstract

Laser-based methods have demonstrated to be effective in the fabrication of surface micro- and nanostructures, which have a wide range of applications, such as cell culture, sensors or controlled wettability. One laser-based technique used for micro- and nanostructuring of surfaces is the formation of laser-induced periodic surface structures (LIPSS). LIPSS are formed upon repetitive irradiation at fluences well below the ablation threshold and in particular, linear structures are formed in the case of irradiation with linearly polarized laser beams. In this work, we report on the simple fabrication of a library of ordered nanostructures in a polymer surface by repeated irradiation using a nanosecond pulsed laser operating in the UV and visible region in order to obtain nanoscale-controlled functionality. By using a combination of pulses at different wavelengths and sequential irradiation with different polarization orientations, it is possible to obtain different geometries of nanostructures, in particular linear gratings, grids and arrays of nanodots. We use this experimental approach to nanostructure the semiconductor polymer poly(3-hexylthiophene) (P3HT) and the ferroelectric copolymer poly[(vinylidenefluoride-co-trifluoroethylene] (P(VDF-TrFE)) since nanogratings in semiconductor polymers, such as P3HT and nanodots, in ferroelectric systems are viewed as systems with potential applications in organic photovoltaics or non-volatile memories.

## 1. Introduction

More and more effort is being made to fabricate functional surfaces with an increased number of applications. In particular, the fabrication of structures in the micro- and nanoscale allows the design of materials with advanced surface properties. Examples of complex structures possessing specific functions and properties can be found in nature [1]. For instance, the control of wettability by the presence of hierarchical surface structures, ranging from micro- to the nanometer scale, may provide self-cleaning properties similar to the lotus leaf in nature [2,3]. Changing and controlling the wetting properties of a material by nanostructuring may allow new applications in the fields of microfluidics, nanofluidics, optofluidics, biomedicine, environmental science and self-cleaning, among others [4,5]. Fabrication of superhydrophobic surfaces by laser irradiation has been reported for different materials [6,7] by the chemical modification of the surface or micro-structuring. Hierarchical structures also play a role in the excellent adhesion of gecko feet [8], and the presence of anisotropic micro- and nanostructures in snake skin affect friction [9].

In order to obtain complex structures, different strategies have been developed based on chemical etching [10,11], lithographic techniques [12,13] or template-based methods [14,15]. Laser-based methods have demonstrated to be effective in the fabrication of surface micro- and nanostructures, which have found a wide range of applications [16]. For example, cell orientation, proliferation and differentiation can be stimulated on biomaterials [17,18,19], bacterial adhesion can be reduced [20,21], wettability may be controlled [22,23,24] and tribological performance [25,26] and superconducting properties may be modified [27]. Some laser-based techniques used for micro- and nanostructuring of surfaces are Direct Laser Interference Patterning (DLIP) [22,28,29] and the formation of laser-induced periodic surface structures (LIPSS) [30,31,32]. LIPSS are formed upon repetitive irradiation at fluences well below the ablation threshold and, in particular, linear structures are formed in the case of irradiation with linearly polarized laser beams, while irradiation with circularly polarized lasers gives rise to the formation of circular nanostructures [30]. The period of the obtained structures is related to the laser wavelength and close to it when irradiation is carried out at normal incidence. During the last 50 years, LIPSS formation has gained attention due to its simplicity and robustness, since it is a single process step which can be carried out in ambient air and is fully compatible with industrial demands in terms of costs, reliability and productivity. Comparing LIPSS with the DLIP set up is simpler, since only one laser beam is employed, and smaller periods may be obtained when high spatial frequency LIPSS (HSFL) are formed [31,33].

Recent research has focused on the functionalization of different materials with structures ranging between a few tens of nanometers up to several micrometers [31,34,35,36]. Substrates nanostructured with LIPSS are proposed to be interesting for applications in different fields, such as optics, electronics, fluidics, sensing, mechanical engineering and biomedicine [16,25,31,37,38,39]. In the case of polymers, most of the research has been focused on the use of nanosecond laser pulses [30], although more recent studies have reported LIPSS formation upon irradiation with picosecond and femtosecond laser pulses [40,41,42]. Some of the applications proposed for the polymer substrates nanostructured in this way are sensors, photovoltaics, non-volatile memories, cell culture and antibacterial substrates [19,21,30,43,44,45,46,47,48].

Additionally, the combination of different laser techniques, such as direct laser writing and DLIP [49] or DLIP with mask imaging [50], may induce the formation of more complex structures, and also multi-pulse DLIP [51], laser micropatterning followed by LIPSS formation [52] or LIPSS simultaneously with diffraction patterns have been reported [53].

In this work, we show how to fabricate a library of ordered nanostructures in a polymer surface by repeated irradiation with a nanosecond laser beam. In particular, we prepared structures on two functional polymers: poly(3-hexylthiophene) (P3HT) and poly(vinylidenefluoride-co-trifluoroethylene) (P(VDF-TrFE)). Nanogratings in semiconductor polymers as P3HT and nanodots in ferroelectric systems are viewed as systems with potential applications in organic photovoltaics [44,54,55] or non-volatile memories [56,57,58]. In the same way that the formation of conventional LIPSS may be induced both in thin polymer films supported on different substrates [59] and in free-standing polymer films [26], the library or ordered structures reported here may be extended to this variety of substrates.

## 2. Materials and Methods

Poly(3-hexylthiophene) (P3HT) thin films were prepared using the spin processor Laurell WS-650 Series. P3HT (purchased from Ossila, Sheffield, UK, batch M102, Mw = 65,200 g/mol, regioregularity 95.7%) powder was dissolved in chloroform (99.98 purity, QUIMIPUR-Spain) with a concentration of 16 g/L. Conductive silicon wafers n-silicon (100, Arsenic dopant, ACM (Lannion, France)) were cut into pieces of 2 cm × 2 cm, cleaned by acetone and 2-propanol, and then dried under nitrogen flow. A fixed volume of 0.2 mL solution was spun coated on silicon wafer at 2400 rpm. Thickness of the polymer films, determined by AFM, was ca. 130 nm.

Additionally, bilayers of P3HT and poly(vinylidene fluoride-trifluoroethylene) P(VDF-TrFE) were prepared. P(VDF-TrFE), purchased from Piezotech S.A.S. (Pierre-Benite, France), with a molar concentration of the random copolymer 76:24 (VDF:TrFE content), Mw = 367,000 g/mol, Mw/Mn = 1.72, was dissolved in methylethylketone (Merck, Darmstadt, Germany) with a concentration of 5 g/L, stirring for 3 h at 70 °C. A fixed volume of 0.2 mL of the P(VDF-TrFE) solution was spun coated on top of the P3HT films. It was previously reported that P3HT is not soluble in methylethylketone [45]. Thickness of the P(VDF-TrFE) film, as determined by AFM, was around 20 nm.

Laser irradiation was carried out in ambient air under normal incidence, with the linearly polarized laser beam of a Q-switched Nd:YAG laser (Lotis TII LS-2131M (Minsk, Belarus), pulse duration 8 ns full width half-maximum) at a repetition rate of 10 Hz. Both the second (532 nm) and the fourth (266 nm) harmonics were used for irradiation. Laser fluence was determined by measuring the laser energy in front of the sample and considering an irradiated area of 5 mm. The total number of pulses and the laser fluence values were chosen on the basis of previous experiments in order to obtain optimal LIPSS [44,60]. Sequential irradiation by changing the laser beam polarization was carried out using a half-wave plate. Large areas were nanostructured by using a sample scanning process. The scanning speed, and consequently, the spatial overlap of successive pulses, was chosen to ensure the delivery of optimal number of pulses for LIPSS formation.

The morphology of the samples was inspected under ambient conditions using a Multimode 8 AFM (Bruker, Karlsruhe, Germany) with a Nanoscope V controller (Bruker, Karlsruhe, Germany). Images were collected in tapping mode using Tap300GHB-G probes (BudgetSensors, Sofia, Bulgaria) and an analysis was carried out using the Nanoscope Analysis software 1.50 (Bruker, Karlsruhe, Germany). Furthermore, in the case of bilayers, piezoresponse force microscopy (PFM) measurements were carried out using the same equipment in the piezoresponse mode. For this, conductive SCM-PIT (Bruker, Karlsruhe, Germany) tips were used. The PFM out-of-plane and in-plane signal was taken applying an AC voltage of 2 V.

Water contact angle (CA) measurements were carried out on the nanostructured films using a pocket goniometer PG2 (FIBRO system, Stockholm, Sweden). The static wetting CA was determined at room temperature and ambient humidity using deionized water.

Grazing incidence small- and wide-angle X-ray scattering (GISAXS and GIWAXS, respectively) were performed by using synchrotron radiation at BL11-NCD-SWEET beamline in ALBA (Cerdanyola del Vallès, Spain). The sample was placed with its surface horizontal and parallel to the X-ray beam and at a height which intercepted half of the beam intensity. Then, the sample was tilted in order to reach an incidence angle of 0.15° between the sample surface and the beam. GISAXS patterns were taken using a PILATUS 1M detector (Dectris, Baden-Daettwil, Switzerland) at 6.612 m from the sample, with exposure times of 5 s. GIWAXS patterns were taken using a LX255-HS detector from Rayonix (Evanston, IL, USA) located at 0.109 m, with exposure times of 10 s.

## 3. Results and Discussion

**Figure 1:**
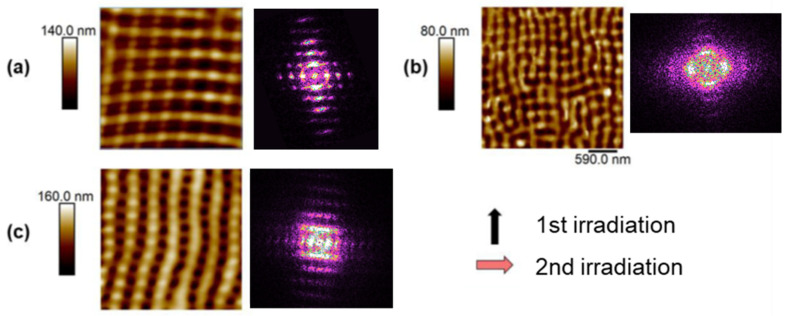
AFM images of combined LIPSS structures formed by sequential irradiation in P3HT. Each AFM image is complemented with its corresponding Fast Fourier Transform (FFT) pattern. (**a**) Sequential irradiation process at λ= 532 nm, 3500 pulses and λ = 532 nm, 100 pulses with the polarization rotated 90°, (**b**) λ = 266 nm, 3300 pulses and λ = 266 nm 300 pulses with the polarization rotated 90° and (**c**) 532 nm, 3600 pulses and λ = 266 nm 300 pulses with the polarization rotated 90°. Arrows indicate the direction of the laser polarization during each irradiation step.

Several experimental parameters have been used to obtain different patterns by laser irradiation at the surface of P3HT films. In particular, number of laser irradiation steps, number of pulses, polarization orientation and laser wavelength have been varied to fabricate different kinds of nanostructures. For example, by performing two consecutive laser irradiations using the same wavelength and rotating the polarization vector 90°, a square-like pattern may be obtained (Figure 1a,b), while if the laser wavelength is changed in the second irradiation step, a rectangle-like pattern will be obtained (Figure 1c).

During a typical process for the generation of gratings obtained by repeated nanosecond pulsed laser irradiation, the polarization direction of the laser is kept constant and the fluence and number of pulses are optimized for each material [30,53,61]. However, in our approach, a selected set of pulses is applied with a different polarization state using a half-wave plate, imposing a new geometry to the pre-existing grating-like one. Since our aim is avoiding ablation of the polymer, the fluence and number of pulses must be similar to the one for which optimum LIPSS gratings were obtained. Thus, we use for both sequential irradiations the laser fluence which gives rise to the formation of well-ordered LIPSS (fluence = 26 mJ·cm^−2^ for irradiations at λ = 532 nm and fluence = 13.4 mJ·cm^−2^ for irradiations at λ = 266 nm) and the total number of pulses is the one that induces the formation of LIPSS upon single irradiation [60].

Figure 1 features atomic force microscopy images of surface nanostructures, forming squares of around 500 nm size (Figure 1a) and of around 200 nm size (Figure 1b), and 500 nm × 200 nm rectangles (Figure 1c). In particular, the image shown in Figure 1a corresponds to a P3HT sample irradiated at 532 nm with 3500 pulses in the first irradiation and 100 pulses in the second irradiation with the polarization shifted 90° with respect to that of the first irradiation. Figure 1b displays the AFM pattern of the surface structures obtained by irradiating a P3HT sample at 266 nm with 3300 pulses in the first irradiation and 300 pulses with 90° shift in the laser polarization in the second. Finally, more complex patterns can be obtained by combining different polarization and different wavelengths. In Figure 1c, a rectangular pattern is formed by a first irradiation at a wavelength of 532 nm and 3600 pulses and afterwards at 266 nm with 300 pulses with a shifted 90° polarization. Besides the real space inspection of the order, possible by the AFM images, the degree of order in the nanostructures can be inferred from the Fourier transformed (FFT) images shown in Figure 1. It can be seen that the FFT image from the sample irradiated at 532 nm at both directions (Figure 1a) shows the intensity maxima along the vertical and horizontal directions. P3HT thin film, irradiated at 266 nm in both directions (Figure 1b), shows a lower order of the LIPSS. Its FFT image depicts two intensity maxima in the horizontal direction and one intensity maximum in the vertical direction. On the other hand, the AFM image from P3HT irradiated with both wavelengths (Figure 1c) shows a clear rectangular structure, corroborated by its FFT image, which shows the intensity maxima along the vertical direction more separated from the origin than the intensity maxima along the horizontal direction.

**Figure 2:**
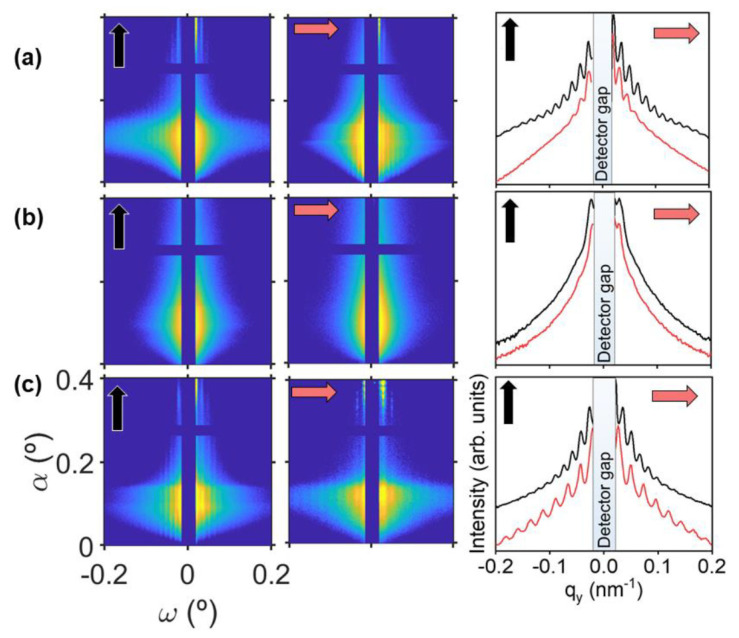
GISAXS patterns from P3HT irradiated samples with (**a**) squares of ~500 nm, (**b**) squares of ~200 nm and (**c**) rectangles of ~500 nm × ~200 nm. Left column: GISAXS patterns taken with the X-ray beam parallel to the first irradiation direction. Center column: GISAXS patterns taken with the X-ray beam parallel to the second irradiation direction. Right column: horizontal cuts from their corresponding GISAXS patterns in the same row, left column (black curves) and center column (red curves). Black arrows indicate the direction of the first irradiation step and red arrows the direction of the second irradiation step. GISAXS patterns taken at incident angle α = 0.4°. Intensity in logarithmic scale.

The quality of the ordered structures can be assessed by GISAXS by placing the sample with the X-ray beam parallel to the polarization of the first irradiation or parallel to the second irradiation. The results are shown also in Figure 2. The GISAXS patterns taken in both orientations are the characteristic ones for one-dimensional paracrystalline structures typical of LIPSS [44,62,63], exhibiting vertical diffraction maxima out of the meridian (ω ≠ 0). In the GISAXS cuts, it is observed that, in the case of squares of ~500 nm (Figure 2a), the array is more ordered along the direction of first irradiation, as revealed by the larger amount of diffraction maxima. The sample with squares of ~200 nm (Figure 2b) shows only one clear diffraction maximum in both directions. The low order in P3HT LIPSS at λ = 266 nm is clearly seen by AFM (Figure 1b), which has already been reported [60]. The sample with rectangles shows a high level of order in both directions (Figure 2c).

**Figure 3:**
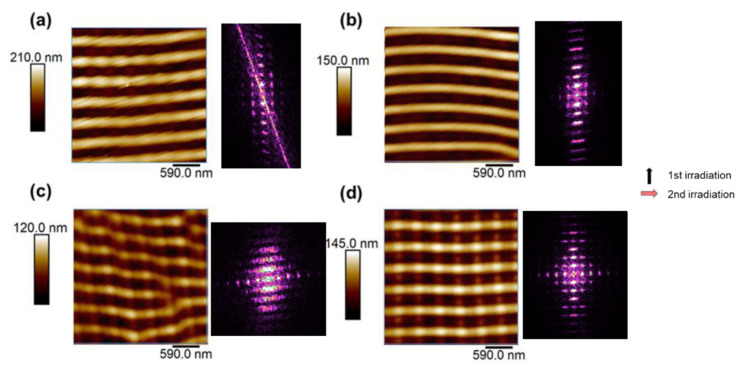
AFM images of combined LIPSS formed by sequential irradiation in P3HT at λ = 532 nm by varying the number of pulses of each irradiation: (**a**) 1800 + 1800 pulses, (**b**) 2400 +1200 pulses, (**c**) 3000 + 600 pulses and (**d**) 3300 + 300 pulses. In the right column, the Fourier Transform of each image is shown. Arrows indicate the direction of the laser polarization during each irradiation step.

Other structures were obtained by using different irradiation conditions. Figure 3 shows AFM images of patterns produced by fixing the wavelength (λ = 532 nm) and polarization shift (90°) and varying the number of pulses, in a way that the total number of pulses is the optimal number to fabricate a conventional P3HT LIPSS (3600 pulses).

It can be seen in Figure 3 that with an equal number of pulses in both irradiation steps, the second polarization direction determines the main direction of the LIPSS. Even with a shorter number of pulses during the second irradiation step (Figure 3c–d), LIPSS are clearly better formed in the direction of the second polarization vector, although FFT patterns indicate that some ordering due to the first irradiation is still present, buried by more perfect LIPSS that appear parallel to the polarization of the second irradiation. Irradiating with 3300 pulses in the vertical direction followed by 300 pulses in the horizontal direction, LIPSS show good order in both directions (Figure 3d), as can be seen in the FFT image, which shows the intensity maxima along both orthogonal directions.

**Figure 4:**
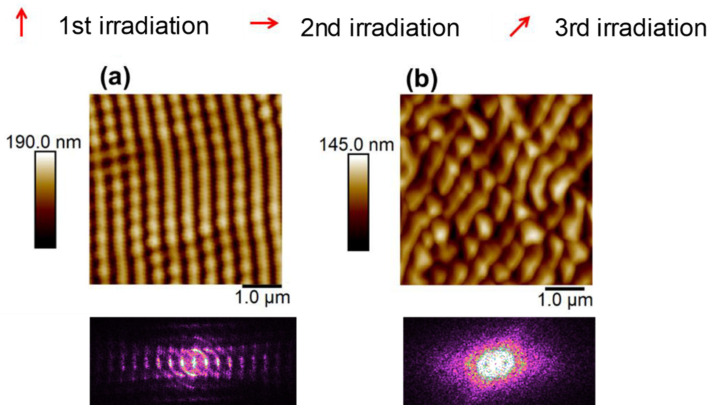
AFM images of combined LIPSS formed by sequential irradiation in P3HT at λ = 532 nm by varying the polarization vector of the laser: (**a**) 3600 + 100 + 100 pulses; (**b**) 3600 + 1800 + 1800 pulses. Next to each AFM figure, its corresponding FFT is shown.

Moreover, more complex structures are fabricated by varying the polarization shifts, number of pulses and adding more irradiation steps. Figure 4 shows the AFM images of these patterns.

Figure 4a shows the AFM image of a P3HT thin film irradiated first with 3600 pulses followed by 100 pulses polarized in the orthogonal direction and 100 pulses polarized 45° with respect to the initial polarization. It can be seen that LIPSS are formed preferentially in the original polarization direction with lower order at 45°, as revealed by several intensity maxima in FFT along the horizontal direction and one maximum along the direction of the polarization used in the third irradiation step. On the other hand, Figure 4b shows an AFM image of the sample irradiated with the same polarization shifts but with 3600 pulses, initially followed by 1800 pulses and then another 1800 pulses. The total number of pulses exceeds the optimum value, so LIPSS hardly preserved a preferential orientation and the corresponding FFT indicates a loss of order compared with Figure 4a. This shows that the polarization used during the last irradiation step will be a determinant to the final order of the sample using a number of pulses about an order of magnitude lower than the number of pulses used during the first irradiation step.

**Figure 5:**
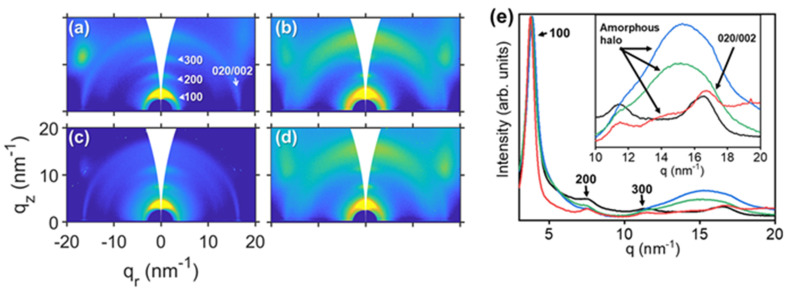
GIWAXS patterns of (**a**) P3HT thin film non-irradiated, and irradiated: (**b**) squares ~500 nm, (**c**) squares ~200 nm, (**d**) rectangles. (**e**) Azimuthal integrated intensity profiles of the GIWAXS pattern: non-irradiated thin film of P3HT (black curve), squares ~500 nm (red curve), squares ~200 nm (blue curve) and rectangles (green curve). GIWAXS of irradiated samples taken with X-ray beam parallel to first irradiation. Inset shows a magnification of the q-range region where the 020/002 reflection and amorphous halo of P3HT appear. Main reflections of crystalline P3HT labeled. Intensity of GIWAXS patterns in logarithmic scale.

Additionally, GIWAXS measurements were carried out to see changes in the inner crystalline structure of LIPSS, in particular whether the crystallinity degree and orientation is affected. Samples with squares of ~500 nm and ~200 nm and rectangles of ~500 nm × ~200 nm were selected and the corresponding GIWAXS patterns are presented in Figure 5.

GIWAXS patterns from Figure 5 show typical P3HT diffraction pattern with preferential “edge-on” orientation, with {h00} reflections oriented along the q_z_ axis. The 020/002 reflection and the halo from the amorphous domains are located around q = 17 nm^−1^. It can be seen that GIWAXS patterns from irradiated samples (Figure 5b–d) present a broad amorphous halo, compared with the pattern from non-irradiated P3HT (Figure 5a).

**Figure 6:**
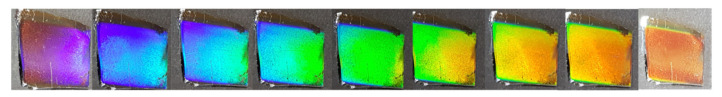
Optical images, obtained under different angles of white light illumination, of 1 × 1 cm^2^ nanostructured samples obtained by using the sequential nanosecond pulsed laser irradiation.

Azimuthal integrations from GIWAXS patterns were done and are represented in Figure 6. It can be observed from the azimuthal integrations (Figure 5f) that the amorphous halo of P3HT increases in the irradiated samples in comparison to the non-irradiated one. Therefore, laser irradiation induces a reduction of the crystallinity degree, as previously reported, as a consequence of rapid heating and cooling processes involved during LIPSS formation [60]. Interestingly enough, the amorphous halo is more prominent in the profiles from samples which have been irradiated at 266 nm in at least one of the irradiation steps: the sample with squares ~200 nm (Figure 5e, blue curve) and the sample with rectangles (Figure 5e, green curve).

Wettability of the nanostructures surfaces was inspected by measuring the water contact angle and the obtained values are cited in Table 1. Initial surface of P3HT is hydrophobic, in agreement with previous results reported in literature [64]. It has been previously reported that laser micro/nanostructuring and in particular LIPSS formation may provoke changes in the wettability of materials due to the morphological changes, to the chemical changes induced upon irradiation or to a combination of both factors. In the present case, after irradiation, differences are not significant, although it seems that CA slightly increases after irradiation at 532 nm and slightly decreases after irradiation at 266 nm. According to the homogeneous wetting model for water, Wenzel’s model [65], the contact between water and the surface of the sample is not altered by the presence of air, and it may explain the variation of the contact angle as a function of ‘r,’ which is given by the relation between the total surface of solid in the solid-liquid interface, and the projection of the total surface of solid in the interface: r = (total surface)/(projected surface), in such a way that the CA of the nanostructured sample is related to the original CA by: cos(CA*) = r·cos(CA). Since LIPSS formation increases ‘r,’ if the original sample is hydrophilic, it will become more hydrophilic, and if hydrophobic, as is the present case, it will become more hydrophobic. Changes in the observed CA may then be explained considering only the morphological changes induced by laser irradiation in the case of 532 nm. As previously reported by some of us [60], an analysis of irradiated P3HT by Near Edge X-ray Absorption Fine Structure (NEXAFS) and Raman spectroscopies revealed good chemical stability after LIPSS formation under laser irradiation conditions similar to the ones used here (same fluence and higher number of pulses). However, in the case of irradiation at 266 nm, we have previously reported that additional modifications are induced upon irradiation, in particular, some photooxidation [66], which could explain the slight decrease of the CA.

The process of obtaining large patterned areas consists of mounting the sample in a translation stage. In this way, areas of several centimeters can be patterned within a few minutes by scanning the sample with the laser beam. For this purpose, the scanning speed, and consequently, the spatial overlap of successive pulses, was chosen to ensure the delivery of optimal number of pulses for LIPSS formation, previously determined by normal single spot irradiation. Nanostructuring in large areas conferred iridescence to the polymer surface. Figure 6 shows a 1 cm × 1 cm piece of P3HT structured with 532 nm squares on a silicon wafer. Pictures are taken by illuminating it with white light at different incident angles and changes in the structural color can be observed. Supplementary Movie 1, recorded with a cellphone, shows the different colors observed as a function of the illumination angle.

**Figure 7:**
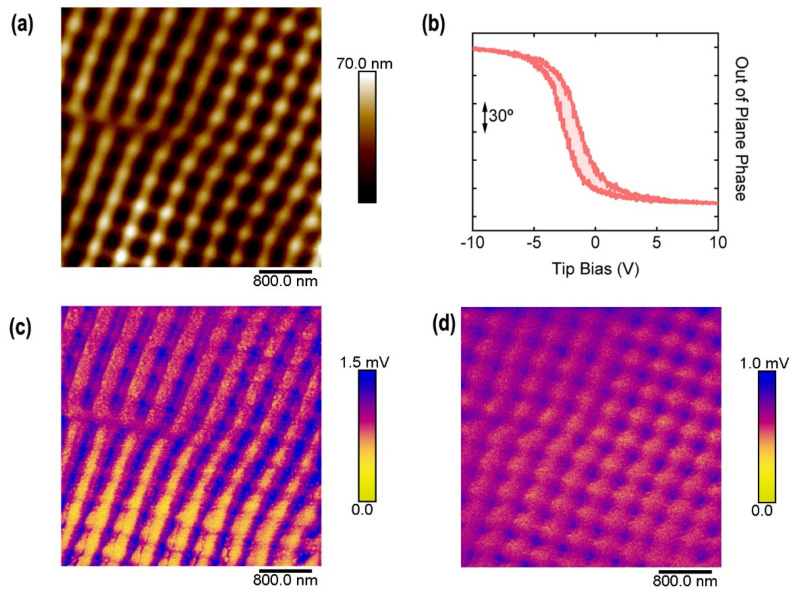
AFM height (**a**) and PFM results (**b**–**d**) of a patterned functional polymer surface exhibiting ferroelectricity. (**b**) Hysteresis of the out-of-plane PFM phase as a function of a DC bias applied to the conducting AFM tip. (**c**) Out-of-plane amplitude mapping of the patterned surface. (**d**) In-plane amplitude mapping of the patterned surface.

**Figure 8:**
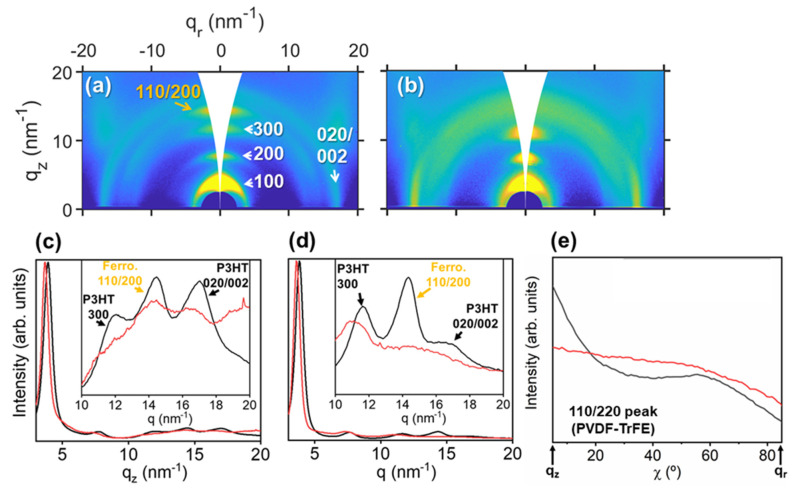
GIWAXS patterns of (**a**) a bilayer of P3HT and P(VDF-TrFE) non-irradiated, and (**b**) bilayer irradiated with two orthogonal polarizations at 532 nm (3500 + 100 pulses). In the right half of (**a**), reflections from P3HT are labeled, and in the left half, the P(VDF-TrFE) reflection is labeled. (**c**) Integration along the q_z_ axis of the non-irradiated bilayer (black curve) and irradiated bilayer (red curve). (**d**) Integration along the whole azimuthal range of the non-irradiated bilayer (black curve) and irradiated bilayer (red curve). Insets in (**c**,**d**) are magnifications of the q-range, where the reflection from P(VDF-TrFE) appears. Reflections from P3HT and P(VDF-TrFE) (ferro.) are labeled in black and orange, respectively. Intensity of GIWAXS patterns in logarithmic scale. (**e**) Radial integration of GIWAXS patterns in the q-range to delimit the 110/200 reflection of P(VDF-TrFE).

This kind of gratings fabricated on functional polymer materials allows nanoscale-controlled functionality. A square pattern was obtained by sequential irradiation at 532 nm (3500 pulses plus 100 pulses after a 90° polarization shift) in a functional polymer bilayer P3HT (bottom)/P(VDF-TrFE) (up). It has been proven in the past that this configuration is suitable in order to obtain nanostructures by laser irradiation on ferroelectric polymers, besides the fact that they are non-absorbers at the used laser wavelength [45]. Figure 7 shows the morphology of the structured bilayer and its piezoelectric nature as revealed by PFM. These results demonstrate that it is possible to obtain an ordered square array of ferroelectric dots. The preserved ferroelectricity in the nanostructured polymer surface is demonstrated by the existence of hysteresis in the out of plane phase signal obtained by piezoresponse force microscopy, although GIWAXS results show an almost completely disappearance of the P(VDF-TrFE) Bragg peak when irradiated (Figure 8).

Figure 8a shows the reflection associated with the (110/200) planes of the ferroelectric phase of P(VDF-TrFE) highly oriented in the q_z_ axis, whereas the GIWAXS pattern from the irradiated bilayer does not show this orientation (Figure 8b). In Figure 8c, it can be seen that the (110/200) reflections from P(VDF-TrFE) along the q_z_ axis is clearly detected in the profile from non-irradiated bilayer (black curve) and it is slightly detected in the profile from the irradiated bilayer (red curve). Looking at Figure 8d, the peak from P(VDF-TrFE) is highly reduced between the profile from the non-irradiated (black curve) and the irradiated bilayer (red curve). Therefore, there is a clear loss of crystallinity in P(VDF-TrFE) when it is irradiated, as well as in irradiated P3HT (Figure 5). However, since PFM results (Figure 7) show piezoelectric behavior, it is supposed that the irradiated P(VDF-TrFE) in the bilayer keeps some degree of crystallinity. Radial integration in the q-range of the (110/002) reflection of P(VDF-TrFE) (Figure 8e) reveals indeed a complete disorientation of a crystalline order that was originally highly oriented in the out-of-plane axis, corresponding with the azimuthal angle χ = 90°, as can be seen in Figure 8a. Therefore, the crystallinity of irradiated P(VDF-TrFE) is not completely removed, but it loses the original preferential orientation that it presents in the non-irradiated bilayer.

## 4. Conclusions

In summary, we reported on the simple fabrication of a library of ordered nanostructures by repeated irradiation using a nanosecond pulsed laser operating in the UV and visible region in order to obtain nanoscale-controlled functionality. As an example, we use this experimental approach to nanostructure a ferroelectric polymer so that an ordered square array of ferroelectric dots is obtained.

## Figures and Tables

**Table 1:** Water contact angle of nanostructured P3HT.

1st Irradiation	2nd Irradiation	3rd Irradiation	Water Contact Angle (°)
Non-irradiated	-	-	96 ± 2
532 nm, 3600 p	-	-	100 ± 3
532 nm, 3500 p	532 nm, 100 p	-	96 ± 1
532 nm, 3300 p	532 nm, 300 p	-	96 ± 3
532 nm, 3000 p	532 nm, 600 p	-	101 ± 4
532 nm, 2400 p	532 nm, 1200 p	-	98 ± 3
532 nm, 1800 p	532 nm, 1800 p	-	98 ± 8
532 nm, 3600 p	532 nm, 100 p	532 nm, 100 p	101 ± 2
266 nm, 3600 p	-	-	86 ± 4
266 nm, 3300 p	266 nm, 300 p	-	72 ± 5
532 nm, 3600 p	266 nm, 300 p	-	89 ± 3

## Supplementary Materials

The following are available online at https://www.mdpi.com/article/10.3390/nano11051123/s1, Video S1: color changes at different angles of white light illumination of nanostructured samples.
